# Supplementary material for: DMRforPairs: identifying Differentially Methylated Regions between unique samples using array based methylation profiles
Source: BMC Bioinformatics. 2014 May 15;15:141. doi: 10.1186/1471-2105-15-141 (PMC4046028; doi:10.1186/1471-2105-15-141)
Supplement: Additional file 2 — DMRforPairs output for the comparison of A431-MCF7 and NA17018-NA17105. Please start from the HTML files in each folder. Available via the BMC Bioinformatics website. [file 1471-2105-15-141-S2.zip › 1394847754114233_MOESM2_ESM/A431_MCF7/figures/8500.pdf]

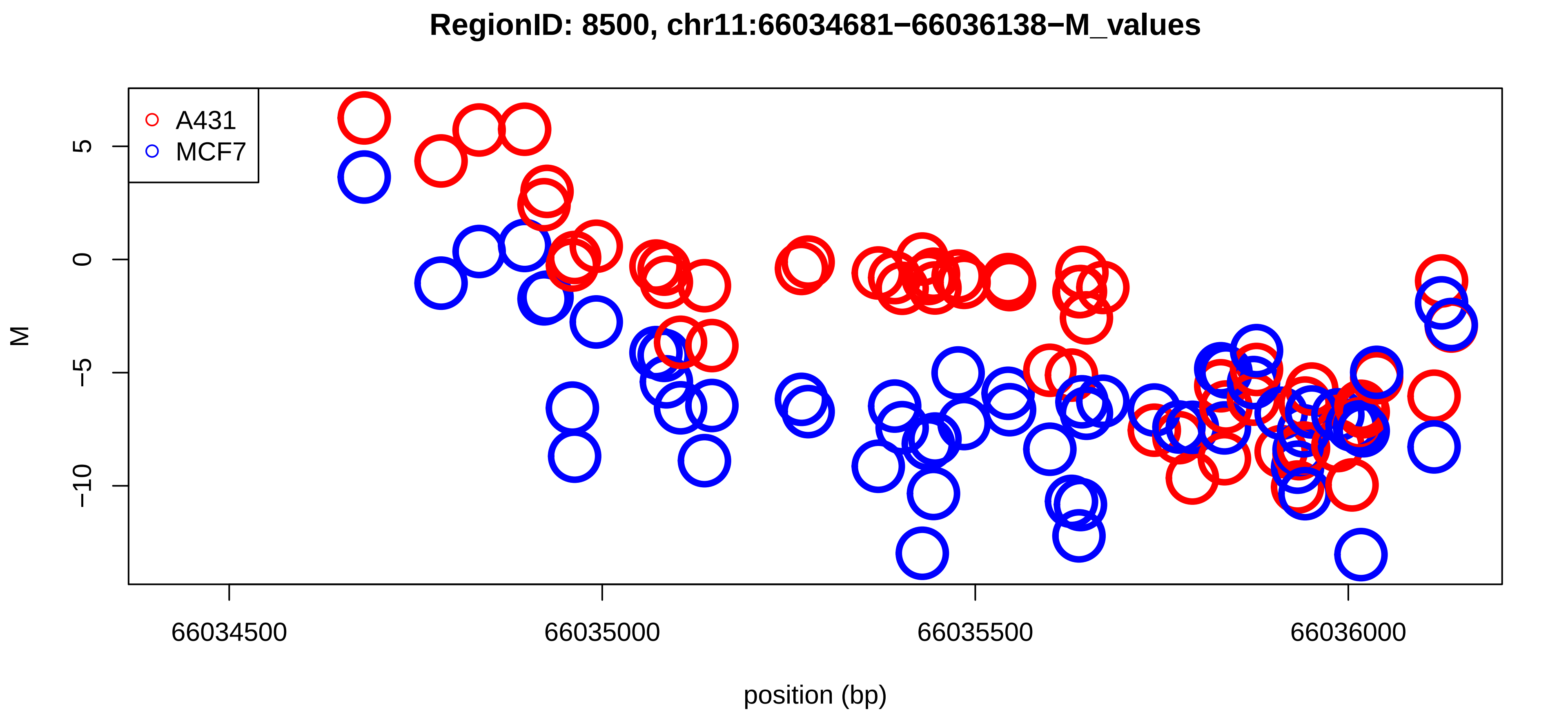

RegionID: 8500, chr11:66034681−66036138−M_values
A431
MCF7
M
5
0
−5
−10
66034500
66035000
66035500
66036000
position (bp)

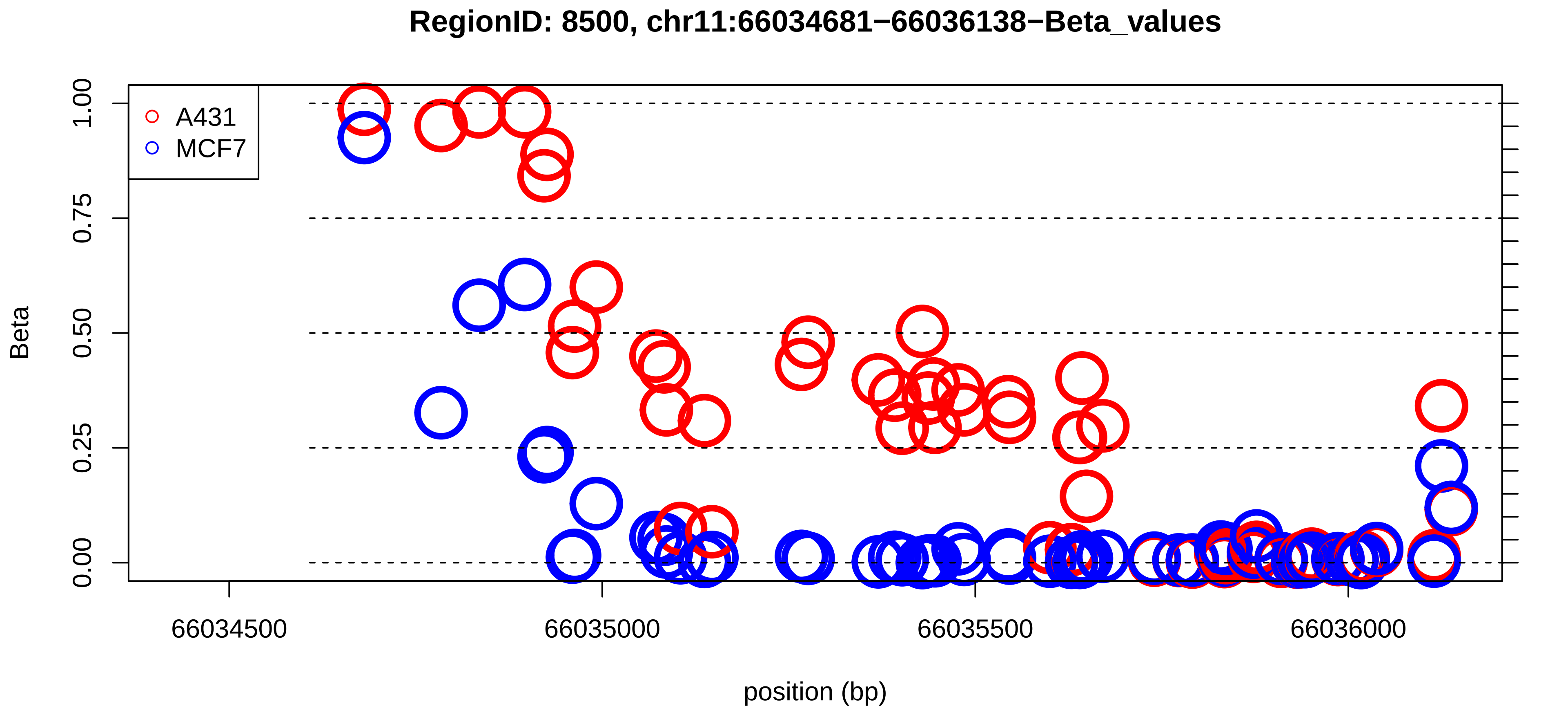

RegionID: 8500, chr11:66034681−66036138−Beta_values
A431
MCF7
Beta
1.00
0.75
0.50
0.25
0.00
66034500
66035000
66035500
66036000
position (bp)

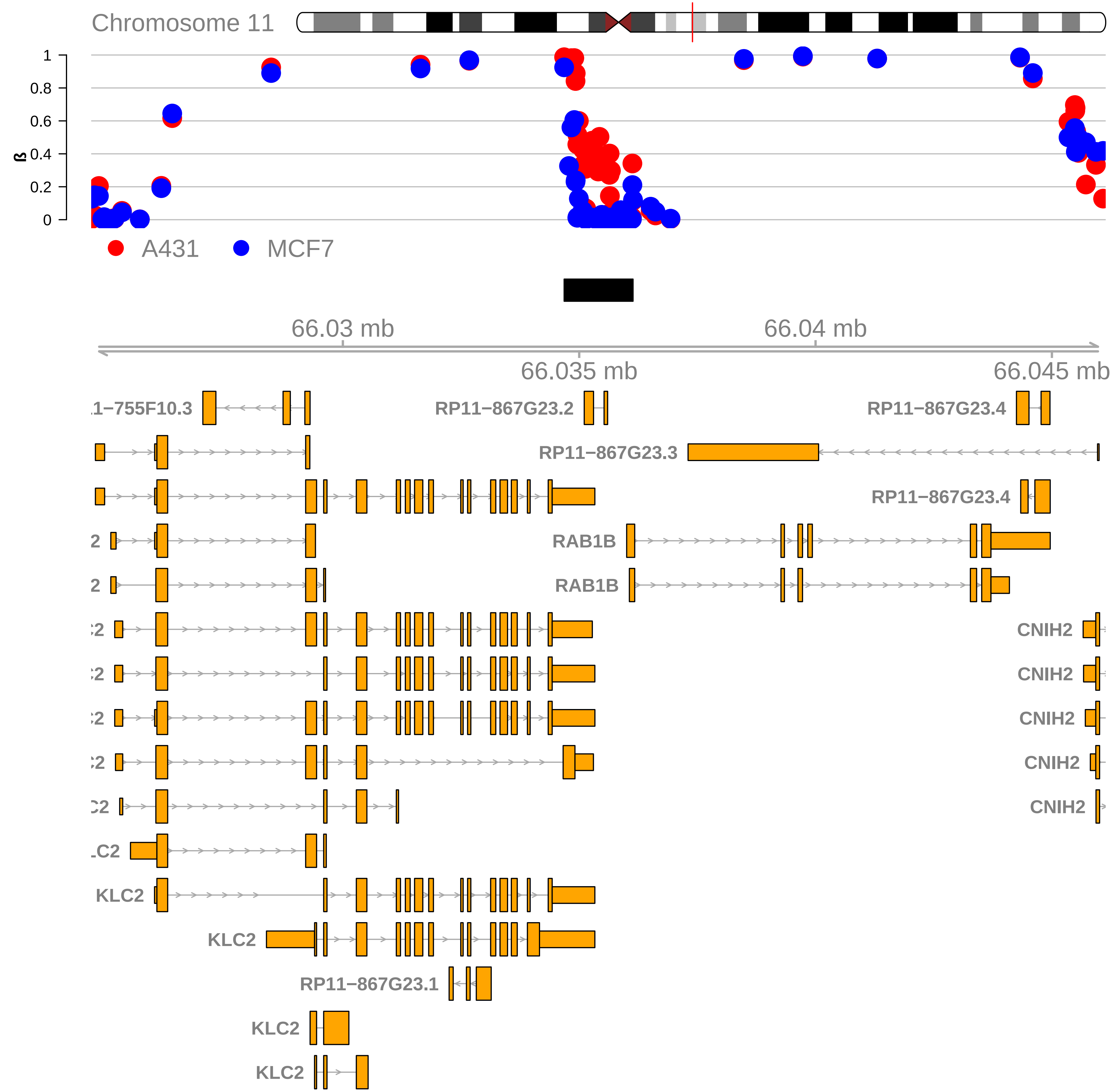

Chromosome 11
1
0.8
0.6
ß 0.4
0.2
0
A431
MCF7
66.03 mb
66.04 mb
66.035 mb
66.045 mb
RP11−867G23.2
RP11−867G23.4
RP11−867G23.3
RAB1B
CNIH2
KLC2
RP11−867G23.1
